# Supplementary material for: The TRACE-Seq method tracks recombination alleles and identifies clonal reconstitution dynamics of gene targeted human hematopoietic stem cells
Source: Nat Commun. 2021 Jan 20;12:472. doi: 10.1038/s41467-020-20792-y (PMC7817666; doi:10.1038/s41467-020-20792-y)
Supplement: Supplementary file 1 — Supplementary Information [file 41467_2020_20792_MOESM1_ESM.pdf]

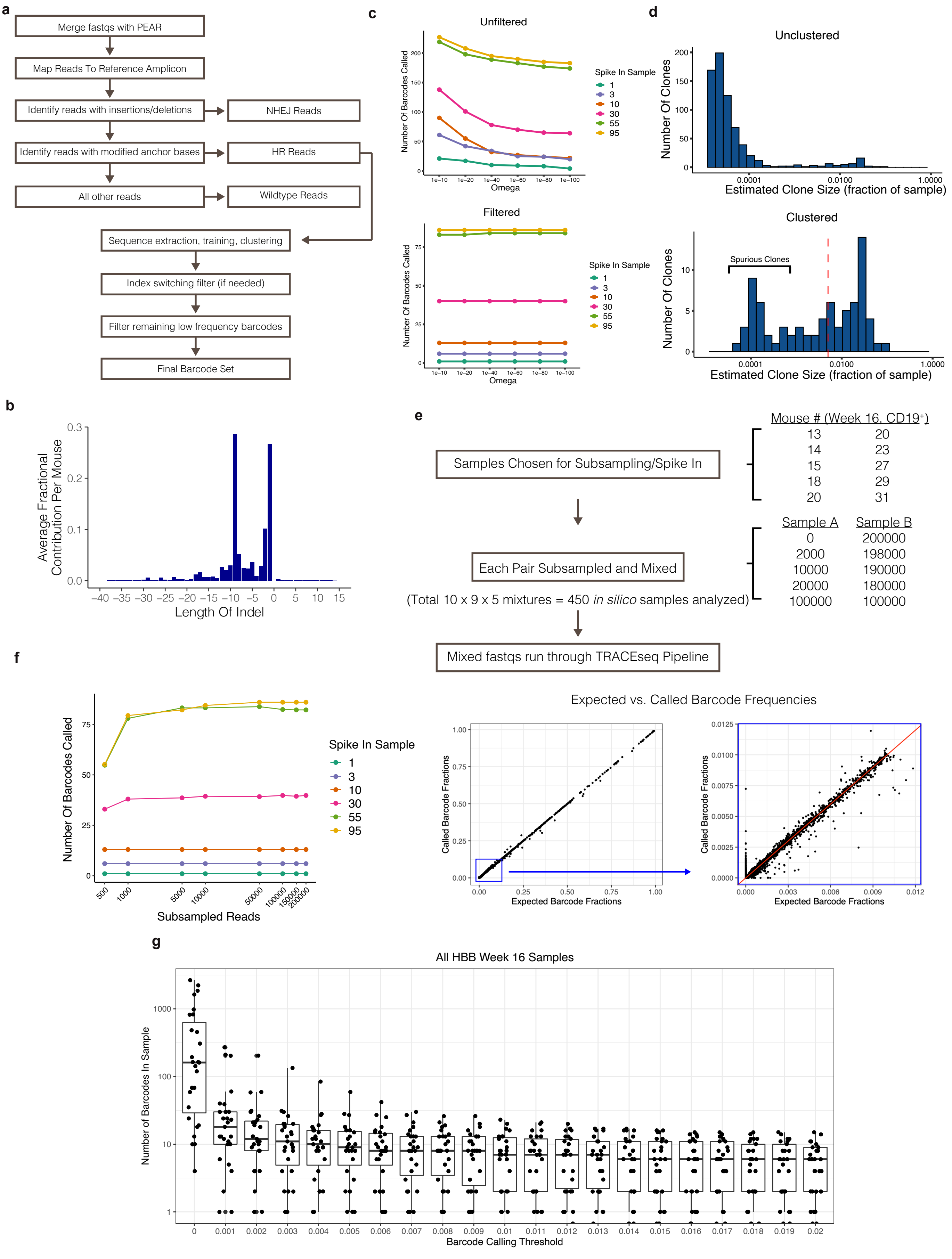

Supplementary Figure 1: Barcode Analysis Pipeline Optimization

Supplementary Figure 1. TRACE-Seq barcode analysis pipeline and optimization. a Schematic of barcode calling pipeline workflow. b Number of barcodes called from sequencing of spike-in samples (**Figure 1e**) as a function of the DADA2 Omega parameter before filtering barcodes <0.5% (top) and after filtering barcodes (bottom). **c** Distribution of barcode sizes for a sample with a known barcode content of 30 barcodes. Top: distribution of unclustered barcodes, showing large numbers of very infrequent sequences on the left side of the distribution. Bottom: distribution of clustered barcodes (bottom). Threshold of 0.5% is depicted by the dashed red line, which was used to enrich for "high confidence" barcodes in subsequent analyses. **d** Subsampling analysis of spike-in samples to estimate necessary sequencing depths to recover the expected numbers of barcodes. **e** In silico mixing of samples used as an alternative method to establish "high confidence" barcode frequencies. Samples mixed at indicated ratios and run through TRACE-seq pipeline. Bottom: Extremely high correlation between expected barcode fractions and results from in silico mixing. **f** Indel frequencies aggregated from all mice analyzed. **g** Total barcodes called as a function of "high confidence" barcode threshold (all Week 16 HBB mice depicted).

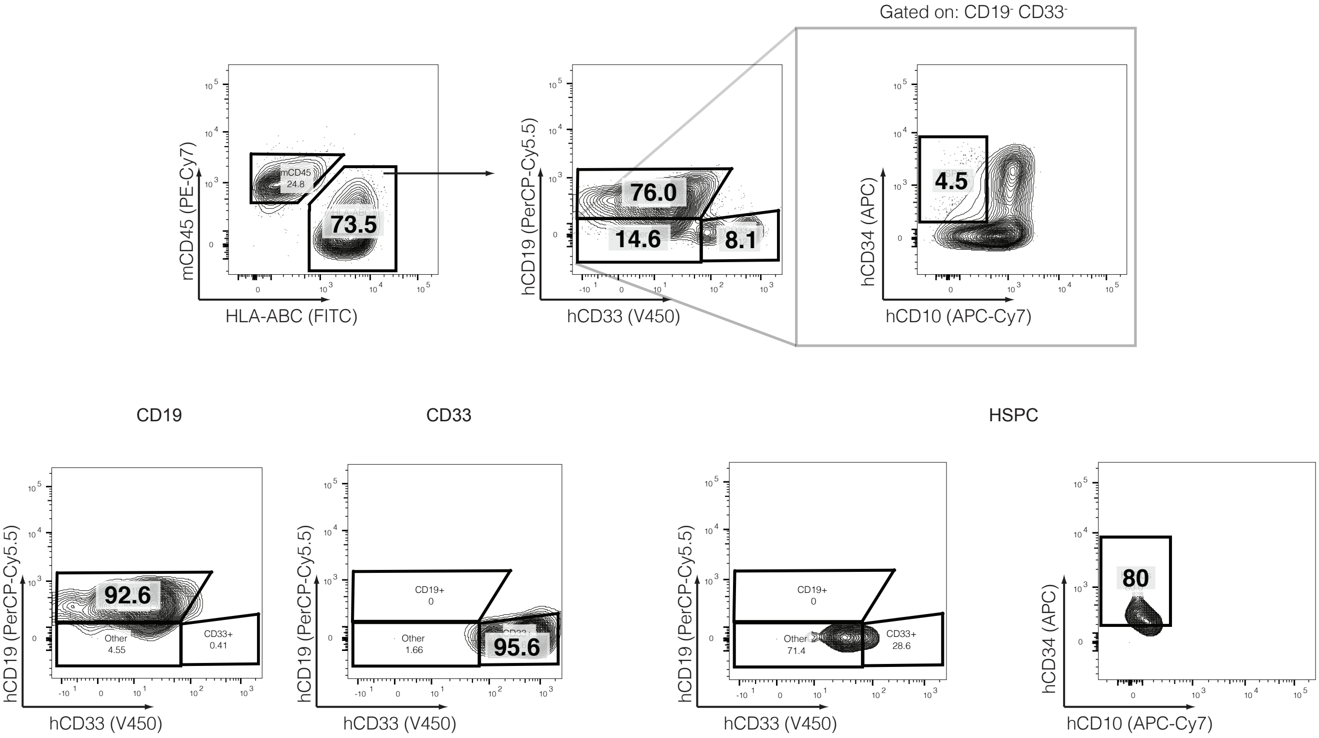

Supplementary Figure 2 Representative HBB gating strategy (upper) and sort purity (lower) for CD19+, CD33+, and HSPCs (CD19-CD33-CD10-CD34+) populations.

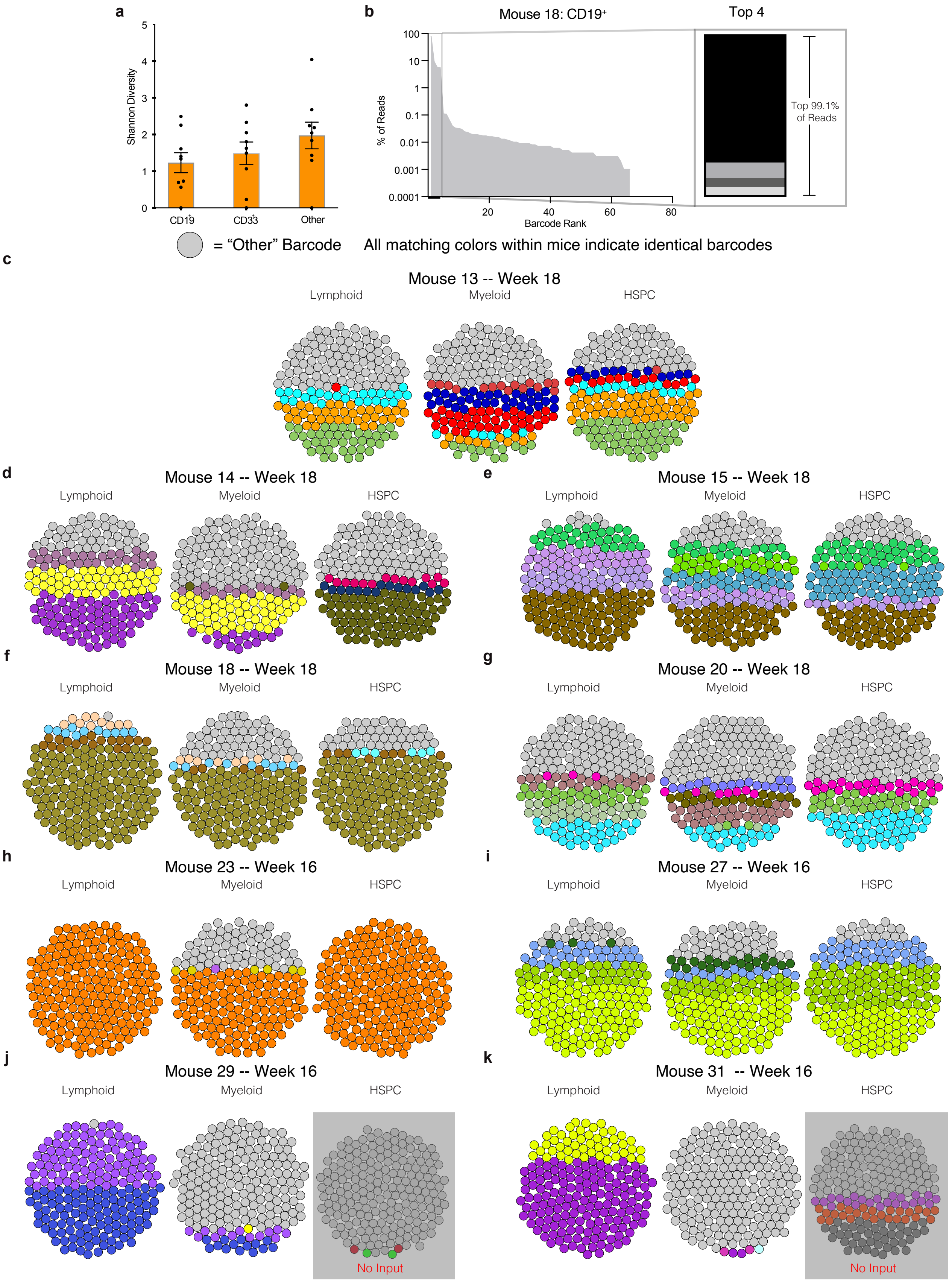

Supplementary Figure 3 Bubble plots depicting shared and unique barcode representation in all HBB barcoded mice at sacrifice

Supplementary Figure 3. Bubble plots depicting shared and unique barcode representation in all HBB barcoded mice at sacrifice. a Simpson diversity, representing barcode richness and evenness, for each lineage. **b** Left: representative quantification of all barcodes in descending order by read proportion. Right: Top four barcodes depicted as stacked bar chart. **c-k** Visualization of top 3 barcodes from all sorted populations (similar to **Figure 4a**) from indicated mice. All other barcodes represented as grey bubbles. Error bars depict mean ± SEM. Note: Despite some colors appearing similar, no top barcodes are shared between distinct mice. See **Supplementary Table 3** for individual HBB sequences and barcode counts.

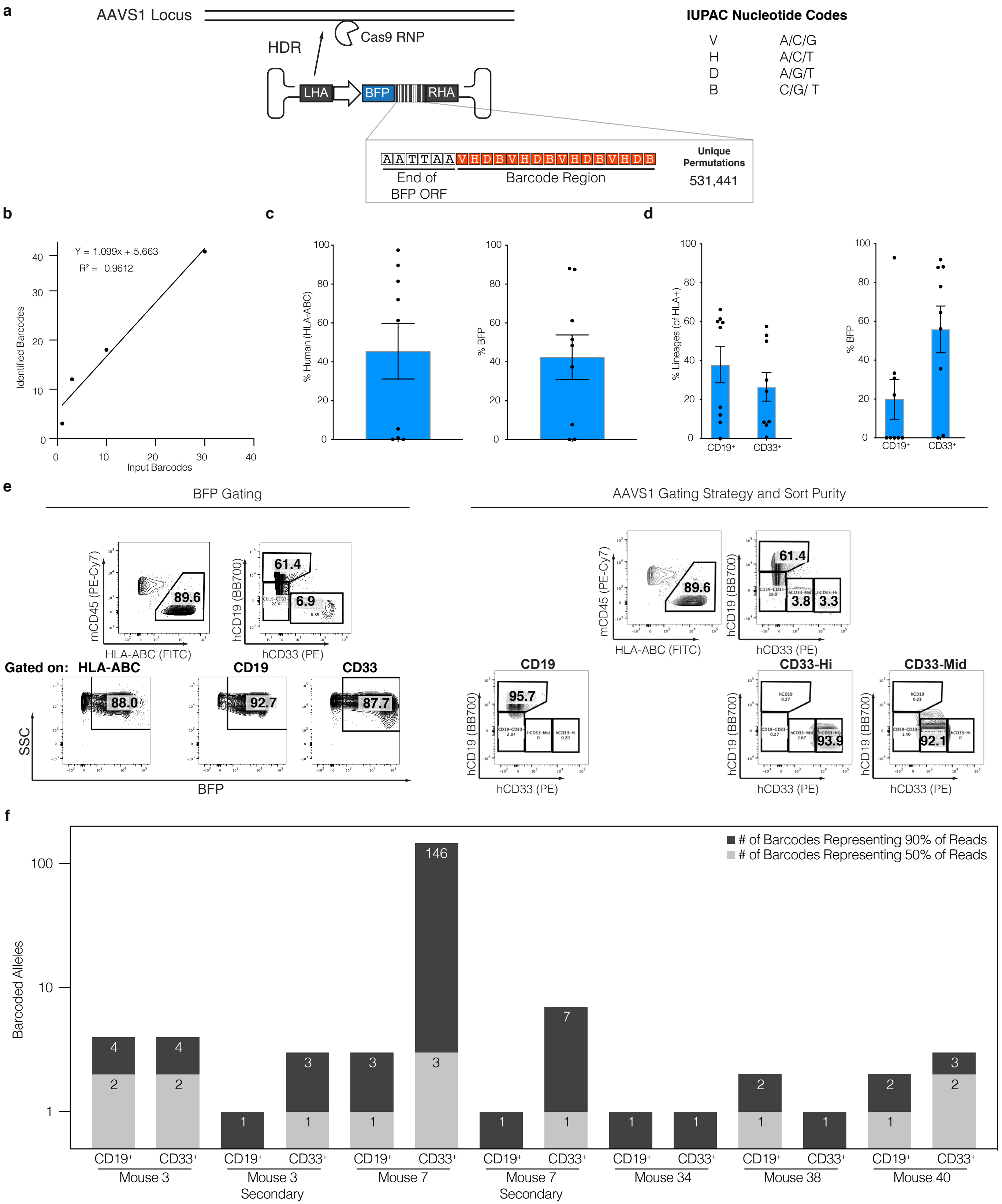

Supplementary Figure 4: Engraftment clonality with genome edited HSPCs is independent of locus and barcode diversity

Supplementary Figure 4. Engraftment clonality with genome edited HSPCs is independent of locus and barcode diversity. a Top: Schema of AAVS1 locus and AAV6 donor containing SFFV-BFP-[barcode]-pA expression cassette. Bottom: Barcode region depicted in red, with a maximum theoretical diversity of 3^{12} = 531,441. **b** Recovery of barcodes from untreated genomic DNA containing 1, 3, 10, and 30 individual plasmids containing BFP barcodes. **c** Total human engraftment in whole bone marrow collected 16-18 weeks post transplantation (expressed as proportion of human HLA-ABC^{+} cells), left, and genome editing efficiency as measured by percentage of BFP+ cells by flow cytometry, right. **d** Multi-lineage engraftment of human CD19^{+} and CD33^{+} lineages, left, with respective genome editing efficiencies, right. Error bars depict mean ± SEM. **e** Example of gating on highly engrafted mouse on %BFP^{+} within each lineage (left) and representative gating strategy and sort purity (right). **f** Barcodes from each subset were sorted from largest to smallest by percentage of reads. Depicted are the numbers of most abundant, unique barcode alleles comprising the top 50% and top 90% of reads from each lineage of all mice transplanted with BC donor edited HSPCs. Mean ± SEM genomes analyzed from each group— CD19^{+}: 7900 ± 1500, CD33^{+}: 10000 ± 3000 (see **Supplementary Table 2**).

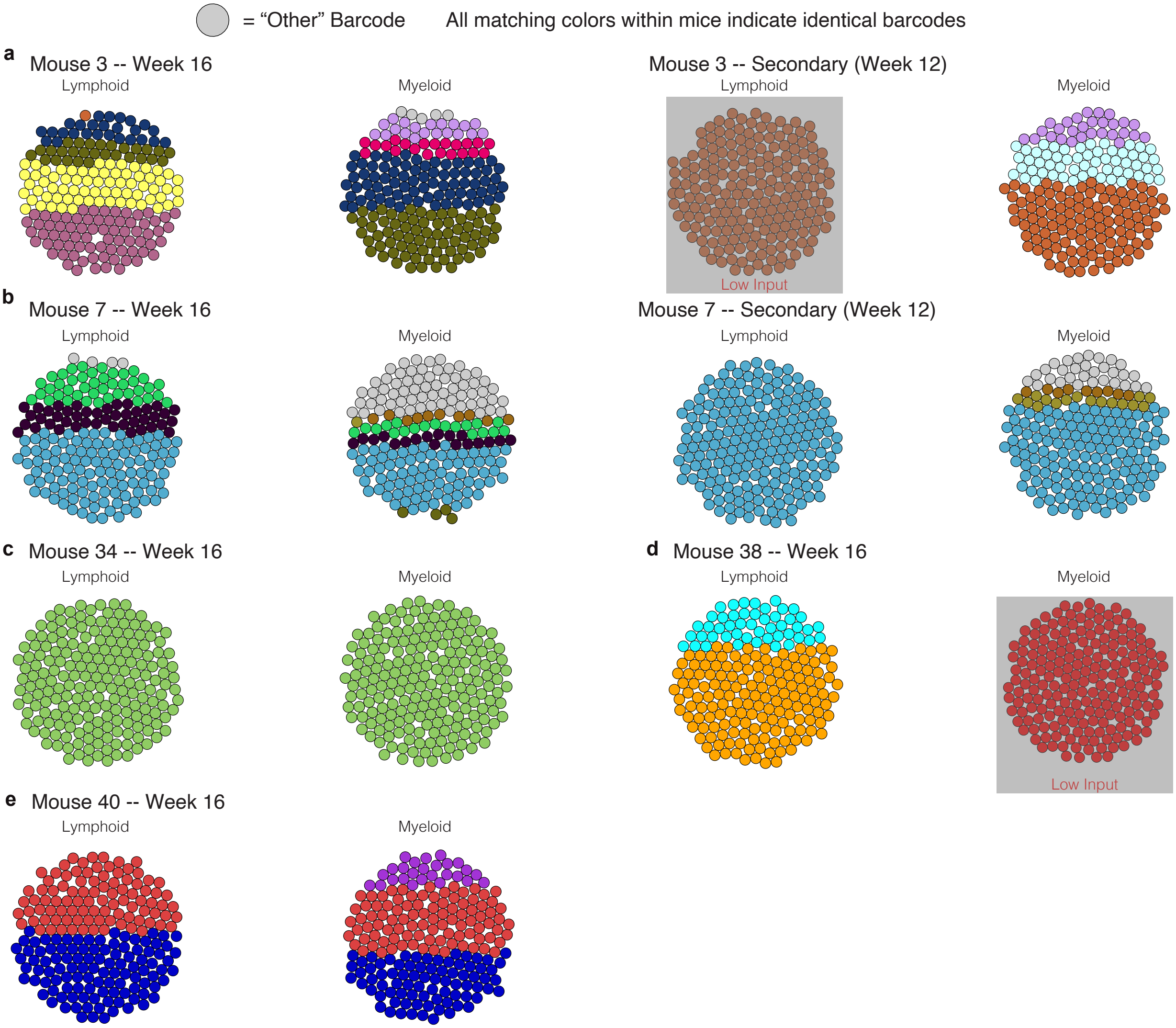

Supplementary Figure 5 Bubble plots depicting shared and unique barcode representation in all BFP barcoded mice at sacrifice. a-e Visualization of top 3 barcodes from sorted populations (similar to **Figure 5a**) from indicated mice. All other barcodes represented as grey bubbles. Despite some colors appearing similar, no top barcodes are shared between distinct mice. See **Supplementary Table 4** for individual BFP sequences and barcode counts.

Supplementary Table 1. HBB barcodes identified in vitro, related to Figure 2c

HBB In Vitro Treatment groups used in HBB in vitro experiments

Biological Replicate	Barcode Pools	Count
1	non-BC	3.30E+05
1	BC1	3.30E+05
1	BC4	3.30E+05
2	non-BC	3.30E+05
2	BC1	3.30E+05
2	BC4	3.30E+05
3	non-BC	2.00E+05
3	BC1	2.00E+05
3	BC2	2.00E+05
3	BC4	2.00E+05
3	BC1/BC2/BC4	2.00E+05
4	non-BC	2.50E+05
4	BC1	2.50E+05
4	BC2	2.50E+05
4	BC4	2.50E+05
5	non-BC	2.50E+05
5	BC1	2.50E+05
5	BC2	2.50E+05
5	BC4	2.50E+05

Supplementary Table 2: HBB Barcodes identified In Vitro (Related to Figure 2c)

Day 2 No BC

HbS	G	C	A	C	C	T	G	A	C	T	C	C	T	G	T	G	G	A	G	A	A	G	T	C	T	Read Percentage	aa_seq	correct_aa	
1	G	C	A	C	C	T	G	A	C	T	C	C	T	G	**A**	G	G	A	**A**	A	A	**A**	T	C	**C**	93.51%	VHLTPEEKSA	TRUE	
2	G	C	A	C	C	T	G	A	C	T	C	C	T	G	T	G	G	A	**A**	A	A	**A**	T	C	**C**	1.89%	VHLTPVEKSA	**FALSE**	**Sickle Allele**
3	G	C	A	C	C	T	G	A	C	T	C	C	T	G	T	G	G	A	G	A	A	G	T	C	T	0.81%	VHLTPVEKSA	**FALSE**	**Sickle Allele**
4	G	C	A	C	C	T	G	A	C	T	C	C	T	G	T	G	G	A	G	A	A	**A**	T	C	**C**	0.46%	VHLTPVEKSA	**FALSE**	**Sickle Allele**
5	G	C	A	C	C	T	G	A	C	T	C	C	T	G	**A**	G	G	A	**A**	A	A	**A**	T	C	**C**	0.33%	AHLTPEEKSA	**FALSE**	
6	G	C	A	C	C	**C**	G	A	C	T	C	C	T	G	**A**	G	G	A	**A**	A	A	**A**	T	C	**C**	0.32%	VHPTPEEKSA	**FALSE**	
7	G	C	A	C	C	T	G	A	C	T	C	C	T	G	T	G	G	A	G	A	A	G	T	C	**C**	0.31%	VHLTPVEKSA	**FALSE**	**Sickle Allele**
8	G	C	**G**	C	C	T	G	A	C	T	C	C	T	G	**A**	G	G	A	**A**	A	A	**A**	T	C	**C**	0.26%	VRLTPEEKSA	**FALSE**	
9	G	C	A	C	C	T	G	A	C	**C**	C	C	T	G	**A**	G	G	A	**A**	A	A	**A**	T	C	**C**	0.25%	VHLTPEEKSA	TRUE	
10	G	C	A	C	C	T	G	A	C	T	C	C	T	G	**A**	G	G	A	**A**	A	A	**A**	T	C	**C**	0.22%	VHLTPEEKSA	TRUE	
11	G	C	A	C	C	T	G	A	C	T	C	C	T	G	**G**	G	G	A	**A**	A	A	**A**	T	C	**C**	0.20%	VHLTPGEKSA	**FALSE**	
12	G	C	A	C	C	T	G	**G**	C	T	C	C	T	G	**A**	G	G	A	**A**	A	A	**A**	T	C	**C**	0.19%	VHLAPEEKSA	**FALSE**	
13	G	C	A	C	C	T	G	A	C	T	C	C	**C**	G	**A**	G	G	A	**A**	A	A	**A**	T	C	**C**	0.18%	VHLTPEEKSA	TRUE	
14	G	C	A	C	C	T	G	A	C	T	C	C	T	G	**A**	G	G	A	**A**	A	A	**A**	T	C	**C**	0.14%	VHLTPEEKSE	**FALSE**	
15	G	C	A	C	C	T	G	A	C	T	C	C	T	G	T	G	G	A	G	A	A	G	T	C	T	0.11%	VHLTPVEKSA	**FALSE**	**Sickle Allele**
16	G	**T**	A	C	C	T	G	A	C	T	C	C	T	G	**A**	G	G	A	**A**	A	A	**A**	T	C	**C**	0.10%	VYLTPEEKSA	**FALSE**	
17	G	C	A	C	C	T	G	A	C	T	C	C	T	G	**A**	G	G	A	**A**	**G**	A	**A**	T	C	**C**	0.09%	VHLTPEEESA	**FALSE**	
18	G	C	A	C	C	T	G	A	C	T	C	C	T	G	**A**	G	G	**G**	**A**	A	A	**A**	T	C	**C**	0.08%	VHLTPEGKSA	**FALSE**	
19	G	C	A	C	C	T	G	A	C	T	C	C	T	G	**A**	G	G	A	**A**	A	A	**A**	T	C	**C**	0.07%	VHLTPEEKST	**FALSE**	

Day 14 No BC

HbS	G	C	A	C	C	T	G	A	C	T	C	C	T	G	T	G	G	A	G	A	A	G	T	C	T	Read Percentage	aa_seq	correct_aa	
1	G	C	A	C	C	T	G	A	C	T	C	C	T	G	**A**	G	G	A	**A**	A	A	**A**	T	C	**C**	94.12%	VHLTPEEKSA	TRUE	
2	G	C	A	C	C	T	G	A	C	T	C	C	T	G	T	G	G	A	**A**	A	A	**A**	T	C	**C**	1.11%	VHLTPVEKSA	**FALSE**	**Sickle Allele**
3	G	C	A	C	C	T	G	A	C	T	C	C	T	G	T	G	G	A	G	A	A	G	T	C	T	1.11%	VHLTPVEKSA	**FALSE**	**Sickle Allele**
4	G	C	**G**	C	C	T	G	A	C	T	C	C	T	G	**A**	G	G	A	**A**	A	A	**A**	T	C	**C**	0.34%	VRLTPEEKSA	**FALSE**	
5	G	C	A	C	C	T	G	A	C	T	C	C	T	G	T	G	G	A	G	A	A	**A**	T	C	**C**	0.32%	VHLTPVEKSA	**FALSE**	**Sickle Allele**
6	G	C	A	C	C	**C**	G	A	C	T	C	C	T	G	**A**	G	G	A	**A**	A	A	**A**	T	C	**C**	0.31%	VHPTPEEKSA	**FALSE**	
7	G	C	A	C	C	T	G	A	C	T	C	C	T	G	**A**	G	G	A	**A**	A	A	**A**	T	C	**C**	0.29%	AHLTPEEKSA	**FALSE**	
8	G	C	A	C	C	T	G	A	C	**C**	C	C	T	G	**A**	G	G	A	**A**	A	A	**A**	T	C	**C**	0.24%	VHLTPEEKSA	TRUE	
9	G	C	A	C	C	T	G	A	C	T	C	C	T	G	**A**	G	G	A	**A**	A	A	**A**	T	C	**C**	0.23%	VHLTPEEKSA	TRUE	
10	G	C	A	C	C	T	G	A	C	T	C	C	T	G	**G**	G	G	A	**A**	A	A	**A**	T	C	**C**	0.23%	VHLTPGEKSA	**FALSE**	
11	G	C	A	C	C	T	G	**G**	C	T	C	C	T	G	**A**	G	G	A	**A**	A	A	**A**	T	C	**C**	0.22%	VHLAPEEKSA	**FALSE**	
12	G	C	A	C	C	T	G	A	C	T	C	C	**C**	G	**A**	G	G	A	**A**	A	A	**A**	T	C	**C**	0.18%	VHLTPEEKSA	TRUE	
13	G	C	A	C	C	T	G	A	C	T	C	C	T	G	T	G	G	A	G	A	A	G	T	C	**C**	0.16%	VHLTPVEKSA	**FALSE**	**Sickle Allele**
14	G	C	A	C	C	T	G	A	C	T	C	C	T	G	**A**	G	G	A	**A**	A	**G**	**A**	T	C	**C**	0.14%	VHLTPEERSA	**FALSE**	

Day 2 BC

HbS	G	C	A	C	C	T	G	A	C	T	C	C	T	G	T	G	G	A	G	A	A	G	T	C	T	Read Percentage	aa_seq	correct_aa	
1	G	C	A	C	C	T	G	A	C	T	C	C	T	G	**A**	G	G	A	**A**	A	A	**A**	T	C	**C**	1.76%	VHLTPEEKSA	TRUE	
2	G	C	A	C	C	T	G	A	C	T	C	C	T	G	T	G	G	A	G	A	A	G	T	C	T	1.31%	VHLTPVEKSA	**FALSE**	**Sickle Allele**
3	**T**	C	A	**T**	**T**	T	**A**	A	C	**A**	C	C	**A**	G	**A**	G	G	A	**A**	A	A	G	T	C	T	0.71%	VHLTPEEKSA	TRUE	
4	G	C	A	**T**	**T**	T	G	A	C	**A**	C	C	T	G	**A**	G	G	A	**A**	A	A	G	T	C	**A**	0.64%	VHLTPEEKSA	TRUE	
5	G	C	A	**T**	**T**	T	G	A	C	T	C	C	**A**	G	**A**	G	G	A	**A**	A	A	**A**	T	C	T	0.37%	VHLTPEEKSA	TRUE	
6	**C**	C	A	**T**	**T**	T	G	A	C	T	C	C	T	G	**A**	G	G	A	G	A	A	G	T	C	**G**	0.35%	VHLTPEEKSA	TRUE	
7	**A**	C	A	**T**	**T**	T	G	A	C	**A**	C	C	T	G	**A**	**A**	G	A	G	A	A	**A**	T	C	T	0.33%	VHLTPEEKSA	TRUE	
8	G	C	A	C	**T**	T	**A**	A	C	**G**	C	C	T	G	**A**	G	G	A	**A**	A	A	**A**	T	C	T	0.32%	VHLTPEEKSA	TRUE	
9	**C**	C	A	**T**	**T**	T	G	A	C	T	C	C	**A**	G	**A**	G	G	A	G	A	A	G	T	C	**A**	0.31%	VHLTPEEKSA	TRUE	
10	G	C	A	**T**	**T**	T	**A**	A	C	T	C	C	T	G	**A**	G	G	A	G	A	A	G	T	C	**C**	0.31%	VHLTPEEKSA	TRUE	
11	**A**	C	A	**T**	**T**	T	**A**	A	C	**A**	C	C	**G**	G	**A**	G	G	A	G	A	A	G	T	C	**C**	0.31%	VHLTPEEKSA	TRUE	
12	**T**	C	A	**T**	**T**	T	**A**	A	C	**C**	C	C	T	G	**A**	G	G	A	G	A	A	G	T	C	**A**	0.31%	VHLTPEEKSA	TRUE	
13	**A**	C	A	**T**	**T**	T	**A**	A	C	T	C	C	T	G	**A**	G	G	A	**A**	A	A	G	T	C	**G**	0.29%	VHLTPEEKSA	TRUE	
14	**C**	C	A	**T**	**T**	T	**A**	A	C	T	C	C	T	G	**A**	G	G	A	G	A	A	**A**	T	C	T	0.28%	VHLTPEEKSA	TRUE	
15	**A**	C	A	**T**	**T**	T	G	A	C	**A**	C	C	**C**	G	**A**	**A**	G	A	G	A	A	G	T	C	**A**	0.28%	VHLTPEEKSA	TRUE	
16	G	C	A	C	**T**	T	G	A	C	T	C	C	T	G	**A**	G	G	A	G	A	A	G	T	C	**C**	0.28%	VHLTPEEKSA	TRUE	
17	**T**	C	A	**T**	**T**	T	G	A	C	T	C	C	T	G	**A**	G	G	A	G	A	A	G	**A**	**G**	T	0.26%	VHLTPEEKSA	TRUE	
18	**C**	C	A	C	**T**	T	**A**	A	C	T	C	C	**A**	G	**A**	G	G	A	G	A	A	**A**	T	C	**C**	0.26%	VHLTPEEKSA	TRUE	
19	**T**	C	A	C	**T**	T	G	A	C	**A**	C	C	T	G	**A**	G	G	A	G	A	A	G	**A**	**G**	**C**	0.26%	VHLTPEEKSA	TRUE	
20	**T**	C	A	**T**	**T**	T	G	A	C	**A**	C	C	T	G	**A**	G	G	A	**A**	A	A	G	T	C	**G**	0.25%	VHLTPEEKSA	TRUE	

Day 14 BC

HbS	G	C	A	C	C	T	G	A	C	T	C	C	T	G	T	G	G	A	G	A	A	G	T	C	T	Read Percentage	aa_seq	correct_aa	
1	G	C	A	C	C	T	G	A	C	T	C	C	T	G	**A**	G	G	A	**A**	A	A	**A**	T	C	**C**	1.50%	VHLTPEEKSA	TRUE	
2	G	C	A	C	C	T	G	A	C	T	C	C	T	G	T	G	G	A	G	A	A	G	T	C	T	0.98%	VHLTPVEKSA	**FALSE**	**Sickle Allele**
3	G	C	A	**T**	C	T	**C**	A	C	**G**	C	C	T	G	**A**	G	G	A	**A**	A	A	G	T	C	T	0.57%	VHLTPEEKSA	TRUE	
4	**T**	C	A	**T**	**T**	T	**A**	A	C	**G**	C	C	**C**	G	**A**	G	G	A	G	A	A	**A**	T	C	T	0.22%	VHLTPEEKSA	TRUE	
5	**C**	C	A	C	**T**	T	**A**	A	C	**G**	C	C	T	G	**A**	G	G	A	**A**	A	A	G	T	C	**A**	0.15%	VHLTPEEKSA	TRUE	
6	**C**	C	A	**T**	C	T	G	A	C	**A**	C	C	T	G	**A**	G	G	A	**A**	A	A	G	**A**	**G**	T	0.15%	VHLTPEEKSA	TRUE	
7	G	C	A	C	**T**	T	**A**	A	C	T	C	C	**G**	G	**A**	G	G	A	G	A	A	G	**A**	**G**	T	0.14%	VHLTPEEKSA	TRUE	
8	**T**	C	A	C	**T**	T	G	A	C	**C**	C	C	T	G	**A**	**A**	G	A	**A**	A	A	G	T	C	**C**	0.14%	VHLTPEEKSA	TRUE	
9	G	C	A	**T**	C	T	**T**	A	C	T	C	C	**G**	G	**A**	G	G	A	G	A	A	**A**	**A**	**G**	**C**	0.13%	VHLTPEEKSA	TRUE	
10	G	C	A	C	C	T	G	A	C	T	C	C	T	G	T	G	G	A	G	A	A	G	T	C	T	0.13%	VHLTPVEKSA	**FALSE**	**Sickle Allele**
11	**T**	C	A	C	**T**	T	G	A	C	T	C	C	T	G	**A**	G	G	A	G	A	A	G	T	C	**C**	0.13%	VHLTPEEKSA	TRUE	
12	**A**	C	A	C	**T**	T	**A**	A	C	**G**	C	C	**C**	G	**A**	G	G	A	G	A	A	G	T	C	**A**	0.13%	VHLTPEEKSA	TRUE	
13	**A**	C	A	**T**	**T**	T	G	A	C	T	C	C	T	G	**A**	**A**	G	A	**A**	A	A	G	**A**	**G**	**C**	0.12%	VHLTPEEKSA	TRUE	
14	**A**	C	A	**T**	**T**	T	G	A	C	**A**	C	C	T	G	**A**	G	G	A	**A**	A	A	G	T	C	**C**	0.12%	VHLTPEEKSA	TRUE	
15	**T**	C	A	**T**	**T**	T	G	A	C	**C**	C	C	**C**	G	**A**	**A**	G	A	G	A	A	**A**	T	C	**G**	0.12%	VHLTPEEKSA	TRUE	
16	G	C	A	**T**	C	T	G	A	C	T	C	C	**G**	G	**A**	G	G	A	**A**	A	A	G	**A**	**G**	T	0.12%	VHLTPEEKSA	TRUE	
17	**C**	C	A	**T**	**T**	T	G	A	C	T	C	C	T	G	**A**	G	G	A	G	A	A	**A**	T	C	**A**	0.12%	VHLTPEEKSA	TRUE	
18	**C**	C	A	**T**	**T**	T	G	A	C	**G**	C	C	T	G	**A**	G	G	A	G	A	A	G	T	C	T	0.12%	VHLTPEEKSA	TRUE	
19	**C**	C	A	**T**	**T**	T	G	A	C	**G**	C	C	T	G	**A**	G	G	A	**A**	A	A	**A**	T	C	**A**	0.12%	VHLTPEEKSA	TRUE	
20	G	C	A	**T**	**T**	T	G	A	C	T	C	C	T	G	**A**	G	G	A	G	A	A	G	T	C	T	0.11%	VHLTPEEKSA	TRUE	

HBB barcodes identified in vitro, related to Figure 2c. Barcodes colorized with red nucleotides indicating differences from unmodified sequence (HbS, top row), along with read percentages, amino acid translation (“aa_seq”), and a True/False value indicating whether the HBB protein translation matches the WT sequence (“correct_aa”).

Supplementary Table 3. Cell counts from each cord blood used for in vivo experiments

Cord Blood	Barcode Pools	Live Count	Viability	N=	Cells/Mouse
1	n/a	4.32E+05	70	1	4.3E+05
2	n/a	3.56E+05	75	2	1.8E+05
3	n/a	9.05E+05	90	2	4.5E+05
4	non-BC	5.94E+05	95	2	3.0E+05
4	BC1 + BC4	1.13E+06	88	2	5.7E+05
5	non-BC	7.13E+05	86	2	3.6E+05
5	BC1 + BC4	8.10E+05	85	2	4.1E+05
6	non-BC	3.24E+05	76	1	3.2E+05
6	BC1 + BC4	4.59E+05	78	1	4.6E+05
7	non-BC	7.60E+05	70	2	2.39E+05
7	BC1/BC2/BC4	1.20E+06	77	2	4.16E+05
8	non-BC	7.80E+05	65	1	4.56E+05
8	BC1/BC2/BC4	6.00E+05	82	1	4.43E+05
9	non-BC	8.00E+05	74	2	2.66E+05
9	BC1/BC2/BC4	7.30E+05	76	2	2.50E+05
9	n/a	1.00E+06	65	2	2.76E+05
10	n/a	6.75E+05	70	2	2.01E+05
11	n/a	4.20E+05	70	1	2.50E+05

Supplementary Table 4. HBB treated mouse BM-MNC subsets sorted as described in Supplementary Figure 2 for each indicated sample, with total sorted events and total genomic input into NGS workflow.

Week	MouseID	Group	Subset	Events Sorted	Genome Equivalents NGS
12	20-Secondary	HBB-BC1/4	HSPC	2316	231.6
12	20-Secondary	HBB-BC1/4	33+	229000	10000
12	20-Secondary	HBB-BC1/4	19+	600000	10000
18	13	HBB-BC1/4	HSPC	40000	4000
18	13	HBB-BC1/4	33+	1000000	10000
18	13	HBB-BC1/4	19+	1000000	10000
18	14	HBB-BC1/4	19+	1000000	10000
18	14	HBB-BC1/4	HSPC	10000	1000
18	14	HBB-BC1/4	33+	444000	10000
18	15	HBB-BC1/4	HSPC	7000	700
18	15	HBB-BC1/4	33+	268000	10000
18	15	HBB-BC1/4	19+	150000	10000
18	18	HBB-BC1/4	19+	400000	10000
18	18	HBB-BC1/4	HSPC	23000	2300
18	18	HBB-BC1/4	33+	500000	10000
18	20	HBB-BC1/4	19+	650000	10000
18	20	HBB-BC1/4	HSPC	15000	1500
18	20	HBB-BC1/4	33+	875000	10000
16	23	HBB-BC1/2/4	HSPC	1250	125
16	23	HBB-BC1/2/4	33+	130000	10000
16	23	HBB-BC1/2/4	19+	45000	4500
16	27	HBB-BC1/2/4	HSPC	5000	500
16	27	HBB-BC1/2/4	33+	390000	10000
16	27	HBB-BC1/2/4	19+	450000	10000
16	29	HBB-BC1/2/4	HSPC	0	0
16	29	HBB-BC1/2/4	33+	30000	3000
16	29	HBB-BC1/2/4	19+	123000	10000
16	31	HBB-BC1/2/4	HSPC	0	0
16	31	HBB-BC1/2/4	33+	50000	5000
16	31	HBB-BC1/2/4	19+	5000	500

Supplementary Table 5: HBB Bubble Plot Detailed Legend

WT	G	C	A	C	C	T	G	A	C	T	C	C	T	G	T	G	G	A	G	A	A	G	T	C	T	translation	correct_aa
1	A	C	A	T	T	T	A	A	C	A	C	C	G	G	A	G	G	A	A	A	A	A	T	C	T	VHLTPEEKSA	TRUE
2	A	C	A	T	C	T	T	A	C	G	C	C	G	G	A	G	G	A	G	A	A	G	A	G	C	VHLTPEEKSA	TRUE
3	A	C	A	C	T	T	A	A	C	G	C	C	A	G	A	A	G	A	G	A	A	A	T	C	G	VHLTPEEKSA	TRUE
4	T	C	A	T	T	T	A	A	C	C	C	C	G	G	A	A	G	A	A	A	A	A	T	C	A	VHLTPEEKSA	TRUE
5	A	C	A	C	C	T	G	A	C	A	C	C	C	G	A	G	G	A	A	A	A	A	A	G	C	VHLTPEEKSA	TRUE
6	G	C	A	T	T	T	G	A	C	C	C	C	A	G	A	G	G	A	A	A	A	A	T	C	G	VHLTPEEKSA	TRUE
7	A	C	A	T	C	T	C	A	C	C	C	C	A	G	A	G	G	A	A	A	A	A	A	G	T	VHLTPEEKSA	TRUE
8	T	C	A	T	T	T	G	A	C	A	C	C	C	G	A	G	G	A	G	A	A	G	T	C	C	VHLTPEEKSA	TRUE
9	T	C	A	T	C	T	T	A	C	G	C	C	T	G	A	A	G	A	G	A	A	G	A	G	T	VHLTPEEKSA	TRUE
10	T	C	A	T	T	T	G	A	C	A	C	C	G	G	A	A	G	A	A	A	A	A	T	C	T	VHLTPEEKSA	TRUE
11	G	C	A	T	C	T	C	A	C	T	C	C	T	G	A	G	G	A	A	A	A	G	A	G	T	VHLTPEEKSA	TRUE
12	G	C	A	C	T	T	G	A	C	G	C	C	G	G	A	A	G	A	G	A	A	G	T	C	A	VHLTPEEKSA	TRUE
13	C	C	A	C	C	T	T	A	C	T	C	C	C	G	A	A	G	A	G	A	A	A	A	G	C	VHLTPEEKSA	TRUE
14	T	C	A	T	C	T	T	A	C	G	C	C	G	G	A	A	G	A	G	A	A	A	A	G	T	VHLTPEEKSA	TRUE
15	T	C	A	C	T	T	A	A	C	T	C	C	C	G	A	G	G	A	A	A	A	A	T	C	C	VHLTPEEKSA	TRUE
16	C	C	A	C	C	T	T	A	C	T	C	C	A	G	A	A	G	A	G	A	A	A	A	G	C	VHLTPEEKSA	TRUE
17	A	C	A	C	C	T	T	A	C	C	C	C	G	G	A	G	G	A	A	A	A	G	A	G	C	VHLTPEEKSA	TRUE
18	A	C	A	T	T	T	G	A	C	T	C	C	C	G	A	A	G	A	G	A	A	A	T	C	A	VHLTPEEKSA	TRUE
19	G	C	A	C	C	T	T	A	C	G	C	C	T	G	A	G	G	A	A	A	A	G	A	G	T	VHLTPEEKSA	TRUE
20	T	C	A	C	T	T	A	A	C	G	C	C	T	G	A	G	G	A	G	A	A	G	T	C	T	VHLTPEEKSA	TRUE
21	G	C	A	C	C	T	G	A	C	T	C	C	T	G	A	A	G	A	A	A	A	A	A	G	C	VHLTPEEKSA	TRUE
22	C	C	A	C	T	T	A	A	C	G	C	C	A	G	A	A	G	A	G	A	A	G	T	C	C	VHLTPEEKSA	TRUE
23	A	C	A	T	C	T	T	A	C	A	C	C	G	G	A	A	G	A	G	A	A	A	T	C	C	VHLTPEEKSA	TRUE
24	C	C	A	C	T	T	G	A	C	C	C	C	T	G	A	G	G	A	A	A	A	G	T	C	T	VHLTPEEKSA	TRUE
25	T	C	A	C	T	T	A	A	C	A	C	C	A	G	A	A	G	A	G	A	A	A	T	C	T	VHLTPEEKSA	TRUE
26	G	C	A	T	T	T	A	A	C	T	C	C	T	G	A	G	G	A	A	A	A	G	T	C	A	VHLTPEEKSA	TRUE
27	C	C	A	T	C	T	C	A	C	T	C	C	G	G	A	G	G	A	A	A	A	A	A	G	T	VHLTPEEKSA	TRUE
28	G	C	A	C	T	T	A	A	C	A	C	C	T	G	A	A	G	A	G	A	A	G	T	C	G	VHLTPEEKSA	TRUE
29	C	C	A	T	C	T	G	A	C	A	C	C	T	G	A	G	G	A	A	A	A	G	A	G	T	VHLTPEEKSA	TRUE
30	G	C	A	T	C	T	A	A	C	T	C	C	T	G	A	A	G	A	A	A	A	A	A	G	T	VHLTPEEKSA	TRUE
31	A	C	A	T	T	T	A	A	C	G	C	C	T	G	A	G	G	A	A	A	A	G	A	G	T	VHLTPEEKSA	TRUE
32	A	C	A	C	T	T	G	A	C	C	C	C	G	G	A	G	G	A	G	A	A	G	A	G	C	VHLTPEEKSA	TRUE
33	G	C	A	T	T	T	G	A	C	A	C	C	A	G	A	G	G	A	G	A	A	A	T	C	T	VHLTPEEKSA	TRUE
34	T	C	A	T	C	T	T	A	C	T	C	C	T	G	A	A	G	A	A	A	A	G	A	G	C	VHLTPEEKSA	TRUE
35	C	C	A	T	T	T	G	A	C	T	C	C	C	G	A	G	G	A	G	A	A	A	T	C	G	VHLTPEEKSA	TRUE
36	C	C	A	C	C	T	A	A	C	T	C	C	A	G	A	G	G	A	A	A	A	G	A	G	C	VHLTPEEKSA	TRUE
37	G	C	A	T	C	T	C	A	C	T	C	C	C	G	A	G	G	A	G	A	A	A	A	G	C	VHLTPEEKSA	TRUE
38	G	C	A	T	T	T	A	A	C	G	C	C	G	G	A	A	G	A	G	A	A	G	T	C	C	VHLTPEEKSA	TRUE
39	G	C	A	T	C	T	G	A	C	T	C	C	T	G	A	G	G	A	G	A	A	G	A	G	T	VHLTPEEKSA	TRUE
40	T	C	A	T	T	T	G	A	C	G	C	C	A	G	A	G	G	A	A	A	A	G	T	C	A	VHLTPEEKSA	TRUE
41	T	C	A	T	T	T	G	A	C	C	C	C	G	G	A	A	G	A	G	A	A	A	T	C	A	VHLTPEEKSA	TRUE
42	T	C	A	T	C	T	T	A	C	A	C	C	G	G	A	G	G	A	A	A	A	A	A	G	T	VHLTPEEKSA	TRUE
43	T	C	A	T	T	T	G	A	C	T	C	C	G	G	A	G	G	A	G	A	A	A	T	C	C	VHLTPEEKSA	TRUE
44	G	C	A	C	C	T	G	A	C	T	C	C	T	G	A	G	G	A	A	A	A	A	T	C	A	VHLTPEEKSA	TRUE
45	G	C	A	T	C	T	G	A	C	T	C	C	T	G	A	G	G	A	G	A	A	G	T	C	T	VHLTPEEKSA	TRUE
46	T	C	A	T	C	T	T	A	C	T	C	C	A	G	A	G	G	A	G	A	A	G	A	G	T	VHLTPEEKSA	TRUE
47	G	C	A	T	C	T	A	A	C	A	C	C	T	G	A	A	G	A	A	A	A	A	T	C	T	VHLTPEEKSA	TRUE
48	G	C	A	T	T	T	A	A	C	C	C	C	C	G	A	G	G	A	G	A	A	G	T	C	C	VHLTPEEKSA	TRUE
49	C	C	A	T	T	T	G	A	C	A	C	C	G	G	A	G	G	A	G	A	A	A	T	C	G	VHLTPEEKSA	TRUE
50	C	C	A	T	T	T	G	A	C	C	C	C	A	G	A	G	G	A	A	A	A	A	T	C	T	VHLTPEEKSA	TRUE
51	G	C	A	T	T	T	G	A	C	G	C	C	T	G	A	G	G	A	G	A	A	G	T	C	A	VHLTPEEKSA	TRUE
52	G	C	A	C	T	T	A	A	C	A	C	C	G	G	A	G	G	A	A	A	A	A	A	G	T	VHLTPEEKSA	TRUE
																				O	T	H	E	R		

		13			14			15			18			20			20-Secondary			23			27			29			31	
WT	19	33	HSPC	19	33	HSPC	19	33	HSPC	19	33	HSPC	19	33	HSPC	19	33	HSPC	19	33	HSPC	19	33	HSPC	19	33	HSPC	19	33	HSPC
1	40	14	49	*	*	*	*	*	*	*	*	*	*	*	*	*	*	*	*	*	*	*	*	*	*	*	*	*	*	*
2	38	11	49	*	*	*	*	*	*	*	*	*	*	*	*	*	*	*	*	*	*	*	*	*	*	*	*	*	*	*
3	27	6	15	*	*	*	*	*	*	*	*	*	*	*	*	*	*	*	*	*	*	*	*	*	*	*	*	*	*	*
4	1	38	14	*	*	*	*	*	*	*	*	*	*	*	*	*	*	*	*	*	*	*	*	*	*	*	*	*	*	*
5	*	34	13	*	*	*	*	*	*	*	*	*	*	*	*	*	*	*	*	*	*	*	*	*	*	*	*	*	*	*
6	*	15	1	*	*	*	*	*	*	*	*	*	*	*	*	*	*	*	*	*	*	*	*	*	*	*	*	*	*	*
7	*	*	*	74	16	*	*	*	*	*	*	*	*	*	*	*	*	*	*	*	*	*	*	*	*	*	*	*	*	*
8	*	*	*	51	58	*	*	*	*	*	*	*	*	*	*	*	*	*	*	*	*	*	*	*	*	*	*	*	*	*
9	*	*	*	30	16	*	*	*	*	*	*	*	*	*	*	*	*	*	*	*	*	*	*	*	*	*	*	*	*	*
10	*	*	*	*	2	78	*	*	*	*	*	*	*	*	*	*	*	*	*	*	*	*	*	*	*	*	*	*	*	*
11	*	*	*	*	*	15	*	*	*	*	*	*	*	*	*	*	*	*	*	*	*	*	*	*	*	*	*	*	*	*
12	*	*	*	*	*	14	*	*	*	*	*	*	*	*	*	*	*	*	*	*	*	*	*	*	*	*	*	*	*	*
13	*	*	*	*	*	*	82	53	51	*	*	*	*	*	*	*	*	*	*	*	*	*	*	*	*	*	*	*	*	*
14	*	*	*	*	*	*	39	16	9	*	*	*	*	*	*	*	*	*	*	*	*	*	*	*	*	*	*	*	*	*
15	*	*	*	*	*	*	36	15	7	*	*	*	*	*	*	*	*	*	*	*	*	*	*	*	*	*	*	*	*	*
16	*	*	*	*	*	*	*	31	60	*	*	*	*	*	*	*	*	*	*	*	*	*	*	*	*	*	*	*	*	*
17	*	*	*	*	*	*	*	27	3	*	*	*	*	*	*	*	*	*	*	*	*	*	*	*	*	*	*	*	*	*
18	*	*	*	*	*	*	32	20	40	*	*	*	*	*	*	*	*	*	*	*	*	*	*	*	*	*	*	*	*	*
19	*	*	*	*	*	*	*	*	*	157	119	142	*	*	*	*	*	*	*	*	*	*	*	*	*	*	*	*	*	*
20	*	*	*	*	*	*	*	*	*	18	5	8	*	*	*	*	*	*	*	*	*	*	*	*	*	*	*	*	*	*
21	*	*	*	*	*	*	*	*	*	11	9	*	*	*	*	*	*	*	*	*	*	*	*	*	*	*	*	*	*	*
22	*	*	*	*	*	*	*	*	*	10	8	*	*	*	*	*	*	*	*	*	*	*	*	*	*	*	*	*	*	*
23	*	*	*	*	*	*	*	*	*	*	*	6	*	*	*	*	*	*	*	*	*	*	*	*	*	*	*	*	*	*
24	*	*	*	*	*	*	*	*	*	*	*	*	31	21	47	*	*	*	*	*	*	*	*	*	*	*	*	*	*	*
25	*	*	*	*	*	*	*	*	*	*	*	*	28	2	2	*	*	*	*	*	*	*	*	*	*	*	*	*	*	*
26	*	*	*	*	*	*	*	*	*	*	*	*	28	2	30	*	*	*	*	*	*	*	*	*	*	*	*	*	*	*
27	*	*	*	*	*	*	*	*	*	*	*	*	21	32	*	192	148	161	*	*	*	*	*	*	*	*	*	*	*	*
28	*	*	*	*	*	*	*	*	*	*	*	*	*	19	*	*	8	*	*	*	*	*	*	*	*	*	*	*	*	*
29	*	*	*	*	*	*	*	*	*	*	*	*	3	7	24	5	4	*	*	*	*	*	*	*	*	*	*	*	*	*
30	*	*	*	*	*	*	*	*	*	*	*	*	*	*	*	*	*	*	199	127	199	*	*	*	*	*	*	*	*	*
31	*	*	*	*	*	*	*	*	*	*	*	*	*	*	*	*	*	*	*	6	*	*	*	*	*	*	*	*	*	*
32	*	*	*	*	*	*	*	*	*	*	*	*	*	*	*	*	*	*	*	1	*	*	*	*	*	*	*	*	*	*
33	*	*	*	*	*	*	*	*	*	*	*	*	*	*	*	*	*	*	*	*	*	107	86	64	*	*	*	*	*	*
34	*	*	*	*	*	*	*	*	*	*	*	*	*	*	*	*	*	*	*	*	*	36	32	78	*	*	*	*	*	*
35	*	*	*	*	*	*	*	*	*	*	*	*	*	*	*	*	*	*	*	*	*	30	16	38	*	*	*	*	*	*
36	*	*	*	*	*	*	*	*	*	*	*	*	*	*	*	*	*	*	*	*	*	3	20	*	*	*	*	*	*	*
37	*	*	*	*	*	*	*	*	*	*	*	*	*	*	*	*	*	*	*	*	*	*	*	*	102	14	*	*	*	*
38	*	*	*	*	*	*	*	*	*	*	*	*	*	*	*	*	*	*	*	*	*	*	*	*	95	13	*	*	*	*
39	*	*	*	*	*	*	*	*	*	*	*	*	*	*	*	*	*	*	*	*	*	*	*	*	*	1	*	*	*	*
40	*	*	*	*	*	*	*	*	*	*	*	*	*	*	*	*	*	*	*	*	*	*	*	*	*	*	2	*	*	*
41	*	*	*	*	*	*	*	*	*	*	*	*	*	*	*	*	*	*	*	*	*	*	*	*	*	*	2	*	*	*
42	*	*	*	*	*	*	*	*	*	*	*	*	*	*	*	*	*	*	*	*	*	*	*	*	*	*	2	*	*	*
43	*	*	*	*	*	*	*	*	*	*	*	*	*	*	*	*	*	*	*	*	*	*	*	*	*	*	*	149	2	*
44	*	*	*	*	*	*	*	*	*	*	*	*	*	*	*	*	*	*	*	*	*	*	*	*	*	*	*	49	*	*
45	*	*	*	*	*	*	*	*	*	*	*	*	*	*	*	*	*	*	*	*	*	*	*	*	*	*	*	*	2	*
46	*	*	*	*	*	*	*	*	*	*	*	*	*	*	*	*	*	*	*	*	*	*	*	*	*	*	*	*	1	*
47	*	*	*	*	*	*	*	*	*	*	*	*	*	*	*	*	*	*	*	*	*	*	*	*	*	*	*	*	*	43
48	*	*	*	*	*	*	*	*	*	*	*	*	*	*	*	*	*	*	*	*	*	*	*	*	*	*	*	*	*	26
49	*	*	*	*	*	*	*	*	*	*	*	*	*	*	*	*	*	*	*	*	*	*	*	*	*	*	*	*	*	23
50	*	*	*	*	*	*	*	*	*	*	*	*	*	1	*	*	2	8	*	*	*	*	*	*	*	*	*	*	*	*
51	*	*	*	*	*	*	*	*	*	*	*	*	*	18	*	*	7	*	*	*	*	*	*	*	*	*	*	*	*	*
52	*	*	*	*	*	*	*	*	*	*	*	*	*	*	*	*	*	5	*	*	*	*	*	*	*	*	*	*	*	*
OTHER	90	79	56	44	105	91	7	34	28	1	56	42	84	93	94	1	27	24	0	64	0	21	42	17	1	168	192	0	193	106

HBB “Bubble plot” detailed legend. Total counts and barcode identities for barcodes depicted in **Figure 4** and **Supplementary Figure 3**.

Supplementary Table 6. AAVS1-BFP treated mouse BM-MNC subsets sorted as described in Supplementary Figure 4

Week	MouseID	Group	Subset	Events Sorted	Genome Equivalents NGS
18	3	AAVS1-BFP-barcode	33-Mid	44000	4400
18	3	AAVS1-BFP-barcode	33-Hi	89000	8900
18	3	AAVS1-BFP-barcode	19+	307000	10000
12	3-Secondary	AAVS1-BFP-barcode	33-Mid	1400	140
12	3-Secondary	AAVS1-BFP-barcode	19+	800	80
12	3-Secondary	AAVS1-BFP-barcode	33-Hi	11500	1150
18	7	AAVS1-BFP-barcode	33-Mid	228000	10000
18	7	AAVS1-BFP-barcode	33-Hi	409000	10000
18	7	AAVS1-BFP-barcode	19+	1166000	10000
12	7-Secondary	AAVS1-BFP-barcode	33-Mid	23300	1553
12	7-Secondary	AAVS1-BFP-barcode	19+	275000	10000
12	7-Secondary	AAVS1-BFP-barcode	33-Hi	65000	4333
16	34	AAVS1-BFP-barcode	33-Mid	56000	5600
16	34	AAVS1-BFP-barcode	33-Hi	61000	6100
16	34	AAVS1-BFP-barcode	19+	1000000	10000
16	38	AAVS1-BFP-barcode	33-Mid	624	62.4
16	38	AAVS1-BFP-barcode	33-Hi	132	13.2
16	38	AAVS1-BFP-barcode	19+	51000	5100
16	40	AAVS1-BFP-barcode	33-Mid	250000	10000
16	40	AAVS1-BFP-barcode	33-Hi	220000	10000
16	40	AAVS1-BFP-barcode	19+	3200000	10000

Total sorted events and total genomic input into NGS workflow. Note that $CD33^{Hi}$ and $CD33^{Mid}$ were combined for analyses.

Supplementary Table 7. BFP Bubble Plot Detailed Legend

	Mouse 3		Mouse 3 Secondary		Mouse 7		Mouse 7 Secondary		Mouse 34		Mouse 38		Mouse 40	
Barcode	19	33	19	33	19	33	19	33	19	33	19	33	19	33
AAGGACGTAAAT	73	*	*	*	*	*	*	*	*	*	*	*	*	*
ACAGATACAAGT	69	*	*	*	*	*	*	*	*	*	*	*	*	*
GTGCGTAGCCGT	28	72	*	*	*	4	*	*	*	*	*	*	*	*
GTTCATGCCCGG	26	77	*	*	*	*	*	*	*	*	*	*	*	*
CCGCATGCCTGT	*	21	*	*	*	*	*	*	*	*	*	*	*	*
CCATAAGCATTC	1	*	200	114	*	*	*	*	*	*	*	*	*	*
ATTGATTTACTG	*	*	*	57	*	*	*	*	*	*	*	*	*	*
CAGGCAATGTGT	*	20	*	28	*	*	*	*	*	*	*	*	*	*
ACTGGAGCCTGT	*	*	*	*	110	79	199	143	*	*	*	*	*	*
GCAGACGCATTG	*	*	*	*	43	18	*	*	*	*	*	*	*	*
CATGACGGGTGG	*	*	*	*	42	19	*	*	*	*	*	*	*	*
GTTCGATGGCGG	*	*	*	*	*	1	*	16	*	*	*	*	*	*
CTTCATGCACTC	*	*	*	*	*	10	*	9	*	*	*	*	*	*
GTAGCAGTGATG	*	*	*	*	*	*	*	*	198	199	*	*	*	*
GCAGCAGTGATG	*	*	*	*	*	*	*	*	*	*	152	*	*	*
ACGTATGGATGC	*	*	*	*	*	*	*	*	*	*	48	*	*	*
GTAGGTGGAAGG	*	*	*	*	*	*	*	*	*	*	*	200	*	*
CAGGGCTTGTGT	*	*	*	*	*	*	*	*	*	*	*	*	106	79
GAAGCCAGAAGG	*	*	*	*	*	*	*	*	*	*	*	*	93	94
AAATGCATGTGT	*	*	*	*	*	*	*	*	*	*	*	*	*	26
OTHER	*	5	*	*	4	64	*	30	*	*	*	*	*	*

BFP "Bubble plot" detailed legend. Total counts and barcode identities for barcodes depicted in **Figure 5** and **Supplementary Figure 5**.
