# Supplementary material for: The TRACE-Seq method tracks recombination alleles and identifies clonal reconstitution dynamics of gene targeted human hematopoietic stem cells
Source: Nat Commun. 2021 Jan 20;12:472. doi: 10.1038/s41467-020-20792-y (PMC7817666; doi:10.1038/s41467-020-20792-y)
Supplement: Supplementary file 2 — Description of Additional Supplementary Files [file 41467_2020_20792_MOESM2_ESM.pdf]

Description of Additional Supplementary Files

Supplementary Data 1. Indel Frequencies - Frequencies of most common indels relative to HBB sgRNA breakpoint.
