# Supplementary material for: The TRACE-Seq method tracks recombination alleles and identifies clonal reconstitution dynamics of gene targeted human hematopoietic stem cells
Source: Nat Commun. 2021 Jan 20;12:472. doi: 10.1038/s41467-020-20792-y (PMC7817666; doi:10.1038/s41467-020-20792-y)
Supplement: Supplementary file 4 — Reporting Summary [file 41467_2020_20792_MOESM4_ESM.pdf]

nature research

Corresponding author(s): Ravindra Majeti

Last updated by author(s): Dec 16, 2019

Reporting Summary

Nature Research wishes to improve the reproducibility of the work that we publish. This form provides structure for consistency and transparency in reporting. For further information on Nature Research policies, see Authors & Referees and the Editorial Policy Checklist .

Statistics

For all statistical analyses, confirm that the following items are present in the figure legend, table legend, main text, or Methods section.

n/a	Confirmed	
☐	☒	The exact sample size (*n*) for each experimental group/condition, given as a discrete number and unit of measurement
☐	☒	A statement on whether measurements were taken from distinct samples or whether the same sample was measured repeatedly
☐	☒	The statistical test(s) used AND whether they are one- or two-sided<br>*Only common tests should be described solely by name; describe more complex techniques in the Methods section.*
☒	☐	A description of all covariates tested
☒	☐	A description of any assumptions or corrections, such as tests of normality and adjustment for multiple comparisons
☐	☒	A full description of the statistical parameters including central tendency (e.g. means) or other basic estimates (e.g. regression coefficient) AND variation (e.g. standard deviation) or associated estimates of uncertainty (e.g. confidence intervals)
☒	☐	For null hypothesis testing, the test statistic (e.g. *F*, *t*, *r*) with confidence intervals, effect sizes, degrees of freedom and *P* value noted<br>*Give P values as exact values whenever suitable.*
☒	☐	For Bayesian analysis, information on the choice of priors and Markov chain Monte Carlo settings
☒	☐	For hierarchical and complex designs, identification of the appropriate level for tests and full reporting of outcomes
☒	☐	Estimates of effect sizes (e.g. Cohen's *d*, Pearson's *r*), indicating how they were calculated

Our web collection on statistics for biologists contains articles on many of the points above.

Software and code

Policy information about availability of computer code

Data collection	Data collection was performed using BD FACSDiva software
Data analysis	Data was analyzed with software including Graphpad Prism, Flowjo, Geneious. Barcode clustering analyses utilized the PEAR aligner, DADA de-noise algorithm and TUBAseq clustering as described in the manuscript

For manuscripts utilizing custom algorithms or software that are central to the research but not yet described in published literature, software must be made available to editors/reviewers. We strongly encourage code deposition in a community repository (e.g. GitHub). See the Nature Research guidelines for submitting code & software for further information.

Data

Policy information about availability of data

All manuscripts must include a data availability statement. This statement should provide the following information, where applicable:

- Accession codes, unique identifiers, or web links for publicly available datasets
- A list of figures that have associated raw data
- A description of any restrictions on data availability

Barcode counts after processing by the TRACEseq pipeline will be made available on the GEO database. Code used to generate bubble plots will be available on github (https://github.com/armonazizi/TRACEseq).

Field-specific reporting

Please select the one below that is the best fit for your research. If you are not sure, read the appropriate sections before making your selection.

Life sciences study design

All studies must disclose on these points even when the disclosure is negative.

Sample size	No sample size calculation was performed.
Data exclusions	No data were excluded from analysis.
Replication	In Vitro experiments were representative of a minimum of three independent biological replicates for each barcode pool. In Vivo experiments represent two independent cohorts of mice, as described in the manuscript.
Randomization	Mice were not randomized to treatment groups.
Blinding	Mouse injections were performed in a blinded manner.

Reporting for specific materials, systems and methods

We require information from authors about some types of materials, experimental systems and methods used in many studies. Here, indicate whether each material, system or method listed is relevant to your study. If you are not sure if a list item applies to your research, read the appropriate section before selecting a response.

Materials & experimental systems

n/a	Involved in the study	
☐	☒	Antibodies
☒	☐	Eukaryotic cell lines
☒	☐	Palaeontology
☐	☒	Animals and other organisms
☒	☐	Human research participants
☒	☐	Clinical data

Methods

n/a	Involved in the study	
☒	☐	ChIP-seq
☐	☒	Flow cytometry
☒	☐	MRI-based neuroimaging

Antibodies

Antibodies used	MNCs were harvested by ficoll gradient centrifugation and human hematopoietic cells were identified by flow cytometry using the following antibody cocktail: HLA-A/B/C FITC (clone W6/32, Biolegend), mouse CD45.1 PE-CY7 (clone A20, Thermo Scientific), CD34 APC (clone 581, Biolegend), CD33 V450 (clone WM53, BD Biosciences), CD19 Percp5.5 (clone HIB19, BD Biosciences), CD10 APC-Cy7 (HI10a, Biolegend), mTer119 PeCy5 (clone Ter-119, Thermo Scientific), and CD235a PE (HIR2, Thermo Scientific). For mice transplanted with AAVS1-edited HSPCs, the following cocktail was used: HLA-A/B/C FITC (clone W6/32, Biolegend), mouse CD45.1 PE-CY7 (clone A20, Thermo Scientific), CD34 APC (clone 581, Biolegend), CD33 PE (clone WM53, BD Biosciences), CD19 BB700 (clone HIB19, BD Biosciences), CD3 APC-Cy7 (clone SK7, BD Biosciences). For AAVS1-edited HSPCs, CD33Hi and CD33Mid were sorted individually, however the data were aggregated for analysis.
Validation	All antibody clones were previously validated and previously published: Bak, R.O., Dever, D.P. & Porteus, M.H. CRISPR/Cas9 genome editing in human hematopoietic stem cells. Nat Protoc 13, 358-376 (2018).

Animals and other organisms

Policy information about studies involving animals; ARRIVE guidelines recommended for reporting animal research

Laboratory animals	6-8 week old NSG female mice were used in the study as described in the methods.
Wild animals	Study did not involve wild animals
Field-collected samples	Study did not involve field collected samples
Ethics oversight	All mouse experiments were conducted in accordance with a protocol approved by the Institutional Animal Care and Use Committee (Stanford Administrative Panel on Laboratory Animal Care no. 22264).

Note that full information on the approval of the study protocol must also be provided in the manuscript.

Flow Cytometry

Plots

Confirm that:

☒ The axis labels state the marker and fluorochrome used (e.g. CD4-FITC).

☒ The axis scales are clearly visible. Include numbers along axes only for bottom left plot of group (a 'group' is an analysis of identical markers).

☒ All plots are contour plots with outliers or pseudocolor plots.

☒ A numerical value for number of cells or percentage (with statistics) is provided.

Methodology

Sample preparation	16-18 weeks following transplantation of targeted HSPCs, mice were euthanized, bones (2x femurs, 2x pelvis, 2x tibia, sternum, spine) were collected and crushed using a mortar and pestle. MNCs were harvested by ficoll gradient centrifugation and human hematopoietic cells were identified by flow cytometry.
Instrument	Samples were analyzed/sorted using a BD FACS Aria II
Software	BD FACSDiva software was used to sort indicated cell populations
Cell population abundance	Post sort purities ranged from 80-95+% as depicted in extended data figures 2 and 4.
Gating strategy	Gating strategies are outlined in extended data figures 2 and 4. Briefly, cells were gated on FSC/SSC, HLA-ABC+ (Human cells), CD19 or CD33 (Human B-lymphocytes or monocytes, respectively), and on CD19-CD33-CD10-CD34+ (Human HSPCs). Cell fractions are expressed as percentage of human mononuclear cells engrafted.

☒ Tick this box to confirm that a figure exemplifying the gating strategy is provided in the Supplementary Information.
